# A pathogenic *UFSP2* variant in an autosomal recessive form of pediatric neurodevelopmental anomalies and epilepsy

**DOI:** 10.1038/s41436-020-01071-z

**Published:** 2021-01-20

**Authors:** Min Ni, Bushra Afroze, Chao Xing, Chunxiao Pan, Yanqiu Shao, Ling Cai, Brandi L. Cantarel, Jimin Pei, Nick V. Grishin, Stacy Hewson, Devon Knight, Sonal Mahida, Donnice Michel, Mark Tarnopolsky, Annapurna Poduri, Alexander Rotenberg, Neal Sondheimer, Ralph J. DeBerardinis

**Affiliations:** 1 Children’s Medical Center Research Institute, UT Southwestern Medical Center, Dallas, TX USA; 2 Department of Pediatrics, UT Southwestern Medical Center, Dallas, TX USA; 3 The Aga Khan University Hospital, Karachi, Pakistan; 4 Eugene McDermott Center for Human Growth and Development, UT Southwestern Medical Center, Dallas, TX USA; 5 Department of Bioinformatics, UT Southwestern Medical Center, Dallas, TX USA; 6 Department of Population and Data Sciences, UT Southwestern Medical Center, Dallas, TX USA; 7 Department of Statistical Science, Southern Methodist University, Dallas, TX USA; 8 Quantitative Biomedical Research Center, UT Southwestern Medical Center, Dallas, TX USA; 9 Bioinformatics Core Facility, UT Southwestern Medical Center, Dallas, TX USA; 10 Department of Biophysics, UT Southwestern Medical Center, Dallas, TX USA; 11 Howard Hughes Medical Institute, UT Southwestern Medical Center, Dallas, TX USA; 12 Division of Clinical and Metabolic Genetics, The Hospital for Sick Children, Toronto, Canada; 13 Department of Neurology, Boston Children’s Hospital and Harvard Medical School, Boston, MA USA; 14 Children’s Medical Center, Dallas, TX USA; 15 Department Pediatrics and Medicine, McMaster University, Hamilton, Ontario Canada

## Abstract

**Purpose:**

Neurodevelopmental disabilities are common and genetically heterogeneous. We identified a homozygous variant in the gene encoding UFM1-specific peptidase 2 (*UFSP2*), which participates in the UFMylation pathway of protein modification. *UFSP2* variants are implicated in autosomal dominant skeletal dysplasias, but not neurodevelopmental disorders. Homozygosity for the variant occurred in eight children from four South Asian families with neurodevelopmental delay and epilepsy. We describe the clinical consequences of this variant and its effect on UFMylation.

**Methods:**

Exome sequencing was used to detect potentially pathogenic variants and identify shared regions of homozygosity. Immunoblotting assessed protein expression and post-translational modifications in patient-derived fibroblasts.

**Results:**

The variant (c.344T>A; p.V115E) is rare and alters a conserved residue in UFSP2. Immunoblotting in patient-derived fibroblasts revealed reduced UFSP2 abundance and increased abundance of UFMylated targets, indicating the variant may impair de-UFMylation rather than UFMylation. Reconstituting patient-derived fibroblasts with wild-type UFSP2 reduced UFMylation marks. Analysis of UFSP2’s structure indicated that variants observed in skeletal disorders localize to the catalytic domain, whereas V115 resides in an N-terminal domain possibly involved in substrate binding.

**Conclusion:**

Different *UFSP2* variants cause markedly different diseases, with homozygosity for V115E causing a severe syndrome of neurodevelopmental disability and epilepsy.

## INTRODUCTION

Disorders of brain development, including those with epilepsy as a prominent feature, are among the most genetically heterogeneous diseases of childhood. OMIM lists over 1,000 Mendelian diseases and disease genes associated with epilepsy, and many others with abnormal brain development and intellectual disability. All known patterns of Mendelian inheritance have been observed in neurodevelopmental disorders and epilepsy, as have imprinting, mitochondrial inheritance, and polygenic effects. Familial aggregation and twin studies have indicated that a genetic cause underlies epilepsy in some 70% of patients.^1,2^ Next-generation sequencing has revolutionized the diagnosis of Mendelian neurodevelopmental disorders and epilepsy, both by enabling massively parallel analysis of known disease genes and by uncovering genes previously unknown to be involved in disorders of brain development.^3,4^

UFmylation is a system of post-translational protein modification similar to ubiquitination in that both pathways use an E1-E2-E3 cascade of reactions.^5^ UFMylation is initiated by cleavage of the ubiquitin-like peptide ubiquitin-fold modifier-1 (UFM1), exposing a glycine residue on UFM1 and rendering it competent for conjugation. This cleavage step is followed by UFM1 adenylation and conjugation to the E1 component, Ubiquitin-like modifier activating enzyme 5 (UBA5).^6,7^ UFM1 is then transferred to the E2 conjugating enzyme UFM1-conjugase 1 (UFC1).^8^ UFM1-conjugated UFC1 and a UFMylation target protein are recruited to the endoplasmic reticulum (ER) membrane by the noncatalytic RING-type E3 component UFM1-ligase 1 (UFL1).^9^ Subsequent, incompletely understood steps lead to mono- or poly-UFMylation of the target protein at the cytosolic face of the ER, followed by release of the UFMylated target into the cytosol.

*UFSP1* and *UFSP2* encode cysteine proteases that cleave UFM1 in the initial step of UFMylation, with UFSP1 performing this cleavage much more efficiently than UFSP2 in vitro.^6^ In addition to their role in producing mature UFM1, both proteases also possess the ability to release UFM1 from UFMylated proteins in a process termed de-UFMylation.^6,10^ The relative importance of UFMylation and de-UFMylation are unknown, and the extent to which UFSP1 and UFSP2 can compensate for each other in vivo has been incompletely explored. Core components of the UFMylation system are conserved throughout metazoans, and loss of components of the UFMylation cycle results in defects in embryogenesis, hematopoiesis, and cellular differentiation in model systems.^5^ However, the precise mechanisms by which UFMylation alters proteostasis are unknown.

In humans, variants in UFSP2’s catalytic domain have been reported in autosomal dominant disorders of the skeletal system, including spondyloepimetaphyseal dysplasia, Di Rocco type (OMIM 617974) and Beukes hip dysplasia in a large Afrikaner family from South Africa (OMIM 142669).^11–13^ These conditions are not reported to cause neurological dysfunction or epilepsy. We report a rare homozygous missense variant in a different UFSP2 domain in four South Asian families with a severe neurological disorder involving intellectual disability, epilepsy, microcephaly, abnormal eye movements, and poor growth.

## MATERIALS AND METHODS

### Patient samples

**Fig. 1:**
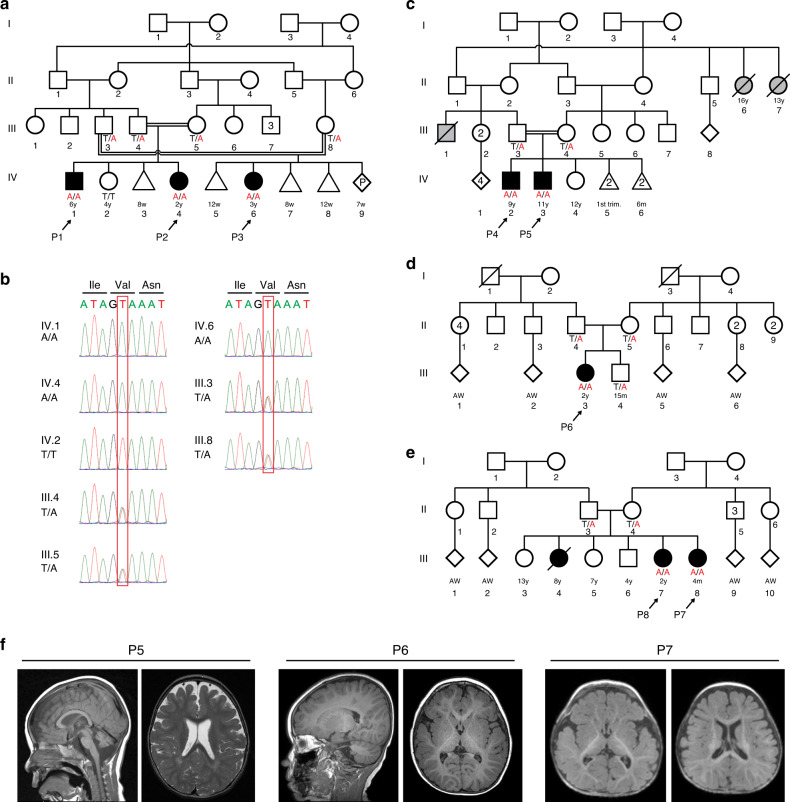
Pedigrees of four unrelated families with affected children carrying the homozygous *UFSP2* p.V115E variant. (**a**) Pedigree showing the relationship of the three affected patients indicated as P1, P2, and P3 from a consanguineous Pakistani kindred (family 1). Genotype annotations show the nucleotide as T for wild-type and A for variant. (**b**) Chromatogram of *UFSP2* sequences confirming parental heterozygosity (III.3, III.4, III.5, and III.8) for c.344T>A, homozygosity for the variant in the three patients (IV.1, IV.4, and IV.6) and homozygous wild-type in the unaffected sister (IV.2). (**c**) Family of patients P4 and P5 (family 2). Three individuals (II.6, II.7, and III.1) who died with unknown neurological disorders are shaded in light gray. (**d**) Family of patient P6 (family 3). AW alive and well. (**e**) Family of patients P7 and P8 (family 4). The female sibling III.4 had a clinically similar disorder and died at 8 years of age. DNA was not available for *UFSP2* sequencing. (**f**) Representative magnetic resonance images (MRIs), including P5, cerebellar volume loss (left) and cortical volume loss (right); P6, mild cerebellar volume loss (left) and mild hypomyelination (right); P7, moderate prominence of cortical cerebrospinal fluid (CSF) space. High-resolution electronic images were not available for P1–P3.

For family 1, whole blood was collected from patients 1–3 and their parents, processed at University of Texas Southwestern Medical Center (UTSW) and subjected to exome sequencing (ES) as described below. Patients 4–8 were analyzed by ES in clinical laboratories as a part of their diagnostic workup at Boston Children’s Hospital (patients 4 and 5), The Hospital for Sick Children in Toronto (patient 6), and McMaster University (patients 7 and 8). Punch biopsies of the skin for fibroblast culture were obtained from patients 1–3 using standard clinical methods. DNA or buccal swab samples were collected from the unaffected siblings in family 1 (IV.2 in Fig. 1a) and family 3 (III.4 in Fig. 1d), respectively.

### Exome sequencing and genomic analysis

Genomic DNAs of the patients and parents from families 1 and 3 were subjected to library preparation using the SureSelect V5 kit (Agilent) following the manufacturer’s instructions. Sequencing was performed on a NovaSeq6000 with 150 bases of paired-end reads to target 200× of raw depth (Psomagen). BWA-MEM was used to align sequence reads to reference genome GRCh37. Post-BAM processing was performed using BWAKit, Samtools 1.4, Sambamba, and GATK 3.7.^14,15^ Variants were detected using GATK 3.7, Platypus,^16^ Samtools version 1.4, and FreeBayes version 0.9.7 (unpublished data). A union VCF file was created with the results from each individual caller for subsequent filtering. The effects of single-nucleotide polymorphisms (SNPs) and indels were predicted using snpEff.^17^ Population frequency was annotated based on gnomAD.^18^ The blood or DNA samples from the patients and parents in families 2, 3, and 4 were submitted to GeneDx for their CLIA-compliant exome sequencing service.

Because of the consanguinity in family 1, the disorder was suspected to be inherited in an autosomal recessive fashion. We therefore filtered for rare missense, nonsense, splicing, or frameshift variants that were homozygous in the patients and heterozygous in the parents, and have a minor allele frequency (MAF) less than 0.01 in gnomAD (v2.1.1; http://gnomad.broadinstitute.org/). Considering the possibility of shared ancestry among the families, ES data from families 1 and 3 were also used to identify runs of homozygosity (ROH) shared by the four affected patients (P1, P2, P3, P6) but not by their unaffected parents using BCFtools/RoH.^19^

### Sanger genotyping

The *UFSP2* variant was confirmed by Sanger sequencing in the three patients and four parents from family 1, and unaffected siblings in families 1 and 3 with genotypes indicated on the pedigrees in Fig. 1. A 415-bp region covering the V115 codon was polymerase chain reaction (PCR) amplified (forward primer: 5’-GGCTGGTCTGAGGGTAGTGA-3’; reverse primer: 5’-TCATTCAAATGTGGCAGTGG-3’). The genotypes were then determined by Sanger sequencing (GENEWIZ).

### Immunoblotting

Whole cell or tissue lysates were extracted using ice-cold TNE buffer (10 mM Tris-Cl, pH 7.5, 1% Nonidet P-40, 150 mM NaCl, 1 mM ethylenediaminetetraacetic acid [EDTA], and protease inhibitors)^10^ followed by three freeze–thaw cycles. The protein supernatants were quantified using the BCA protein assay (Pierce, 23227). Proteins were separated on 4–20% sodium dodecyl sulfate polyacrylamide gel electrophoresis (SDS-PAGE) gels (Bio-Rad), transferred to PVDF membranes, and probed with primary antibodies against the indicted proteins, including anti-UFSP2 (Proteintech, 16999-1-AP), anti-UFM1 (Abcam, ab109305), anti-DDRGK1 (Proteintech, 21445-1-AP), anti-TRIP4 (Proteintech, 12324-1-AP), anti-RPL26 (Cell Signaling, 2065), anti-BiP (Cell Signaling, 3177), anti-XBP1s (Cell Signaling, 12782), anti-GAPDH (Cell Signaling, 8884), anti-UFSP1 (Santa Cruz Biotechnology, sc-398577) and anti-Calnexin (Enzo Life Sciences, ADI-SPA-860-F) antibodies. Immunoreactive proteins were visualized by enhanced chemiluminescence (Pierce, 32106). Signal intensities for the anti-UFM1 were quantified using ImageJ. In Fig. 3b, the error bars are the SD for the same three control and three patient samples on the blot in Fig. 3a, and a two-tailed unpaired *t*-test was used to compare the groups.

### Quantitative real-time PCR

Total RNA was isolated from 6×10^5^ fibroblasts using the RNeasy minikit (Qiagen) according to the manufacturer’s protocol. 1 μg of RNA was used for complementary DNA (cDNA) synthesis using with the iScript™ cDNA Synthesis Kit (Bio-Rad). Quantitative real-time (RT-PCR) was performed using the SYBR Green mix (Bio-Rad). The *UFSP2* cDNA was PCR amplified using forward primer 5’-GTTATGATCGGGGGAGGAGT-3’ and reverse primer 5’-CAGGTCTTCAGCACCGGTAT-3’. In Fig. 3c, the data are the average and SD for three technical replicates from each cell line. Data between the controls and patients were compared by unpaired *t*-tests and found not to differ significantly.

### Molecular cloning, lentiviral production, and transduction

Wild-type and V115E-mutant open reading frames (ORFs) of human UFSP2 were PCR amplified from the cDNA pool of HEK293T cells or the fibroblasts of patient P1, respectively. We used the following primers for PCR amplification: 5’-AGATCTGCCGCCGCGATGGTGATTTCAGAAAGTATGGAT-3’ (forward) and 5’-GCGGCCGCGTACGCGAATCATATTTGGTCGCTGAGGA-3’ (reverse). The fragments were purified and cloned into pLenti-EF1a-C-Myc-DDK-IRES-Puro (OriGene) using the In-Fusion HD Cloning Kit following the manufacturer’s instructions (Takara Bio). The positive clones were confirmed by Sanger sequencing (GENEWIZ). Lentivirus was produced by transfecting HEK293T cells using lipofectamine 3000 reagent (Thermo Fisher Scientific). Viral supernatants were harvested at 48 hours and 72 hours, filtered through a 0.45-μm filter, and concentrated using PEG-it Virus Precipitation Solution (System Biosciences). For transduction, the lentiviral pellets were suspended in culture medium and added to fibroblasts at 70–80% confluency in 6-well plates. After 48 hours of transduction, the fibroblasts were selected under puromycin for one week for stable expression of wild-type or mutant UFSP2.

## RESULTS

### Homozygous *UFSP2* missense variant in a severe, early-onset neurological disorder

Clinical features of the patients are summarized in Table 1. Patients 1–3 are from a consanguineous family (family 1) in Pakistan whose four-generation pedigree is shown in Fig. 1a. Patients 1 (IV.1) and 2 (IV.4) were born to first-cousin parents, and patient 3 (IV.6) was born to a different set of first-cousin parents within the same kindred. Two of these children were at or below the 5th percentile for weight at birth, and all three have displayed poor postnatal weight gain. Two patients are microcephalic. All three had early-onset, generalized epilepsy and nonparalytic convergent strabismus. All patients have marked developmental impairments. At age 3, patient 3 (IV.6) can toe-walk with assistance, smile, engage in simple nonverbal communications (e.g., tapping her head), and feed herself finger foods. The other two patients are hypotonic with minimal head control, no walking, and essentially no communication beyond occasional vocalizations. A brain magnetic resonance image (MRI) was performed in patient 1 (IV.1) and revealed bilateral thinning of the deep periventricular white matter and cerebellar hypoplasia.

**Table 1:** Clinical characteristics of 8 patients homozygous for the V115E variant in *UFSP2*.

	Patient 1	Patient 2	Patient 3	Patient 4	Patient 5	Patient 6	Patient 7	Patient 8
**Demographics**								
Sex	Male	Female	Female	Male	Male	Female	Female	Female
Current age, years	6	2	3	9	11	2	1	2.5
Country	Pakistan	Pakistan	Pakistan	Pakistan	Pakistan	Afghanistan	Afghanistan	Afghanistan
**Birth history**								
Gestational age	36 weeks	36 weeks	36 weeks	39 weeks	42 weeks	41 weeks	40 weeks	40 weeks
Weight at birth, kg (percentile)^a^	2.5 kg (5th)	3.5 kg (58th)	2 kg (1st)	3.18 kg (27th)	4.43 kg (97th)	3 kg (21st)	3.23 kg (36th)	NA
**Anthropometry**								
Weight, age (percentile)^a^	10.4 kg, 6 years (<3rd)	6.1 kg, 2 years (<3rd)	11.9 kg, 3 years (<3rd)	24.4 kg, 8.5 years (5–10th)	27.8 kg, 11 years (5–10th)	9.8 kg, 2 years (<3rd)	7.3 kg, 10 months (3–5th%)	8.3 kg, 14 months (3rd)
Height, age (percentile)^a^	104 cm, 6 years (<3rd)	76.5 cm, 2 years (<3rd)	93 cm, 8 years (25th)	118 cm, 8.5 years (<3rd)	129 cm, 11 years (<3rd)	84.5 cm, 2 years (25–50th)	NA	NA
OFC, age (percentile)^a^	48.9 cm, 6 years (3–15th)	41.5 cm, 2 years (<3rd)	45.5 cm, 3 years (<3rd)	47.5 cm, 3.5 years (10th)	49 cm, 7 years (3–15th)	42 cm, 2 years (<3rd)	44 cm, 10 months (40th)	45 cm, 22 months (5th)
**Phenotype**								
Initial symptom (age)	Seizures (5 months)	Seizures (2 days)	Seizures (7 months)	Seizures (3.5 months)	Seizures (3 months)	Seizures (3 months)	Seizures (3 months)	Seizures (3 months)
Epilepsy type	Generalized	Generalized	Generalized	Generalized	Generalized	Infantile spasms	Generalized	Generalized
Seizure type at onset	Tonic clonic	Focal clonic	Tonic clonic	Tonic	Infantile spasms	Infantile spasms	Infantile spasms	Infantile spasms
Seizure frequency	10–15/day	12–15/day	3–4/day, mostly during sleep	~15/day (seizure-free × 2.5 years)	1–7/month	3–4/day	~ 3/day, clusters of 10	~ 2/day, clusters of 10
Cognition	ID	ID	ID	ID	ID	ID	ID	ID
Speech	Occasionally vocalize	Occasionally vocalize	Occasionally vocalize	Nonverbal	Nonverbal	Nonverbal	Random cooing	No sounds
Tone	Hypotonia	Hypotonia	Normal	Hypotonia	Hypotonia	Increased in limbs	Hypotonia	Hypotonia
Eyes	B/L nonparalytic convergent squint	B/L nonparalytic convergent squint	B/L nonparalytic convergent squint	Alternating esotropia	Exotropia	Esotropia, but tracks	Normal	Normal

Birth OFCs were not documented for any of these patients.

*OFC* occipital frontal circumference, *ID* intellectual disability, *NA* not available, *B/L* bilateral.

^a^Growth data from the Centers for Disease Control and Prevention were used to calculate percentiles, except for head circumferences in children older than 3 years, in which case charts from the World Health Organization were used.

ES was performed in the three patients and their parents in family 1. Only one missense variant in *UFSP2* (NC_000004: g.186337011A>T; NM_018359: c.344T>A; NP_060829: p.V115E; rs142500730) passed the filtering criteria. All three patients were homozygous and their unaffected parents were heterozygous. The ES results were confirmed by Sanger sequencing, which also demonstrated that the unaffected sibling IV.2 is homozygous for the wild-type sequence (Fig. 1b). We submitted this variant to ClinVar with the accession number of SCV001338803.

Depositing this variant together with phenotypic information into GeneMatcher (https://genematcher.org) led to the identification of five additional homozygotes from Pakistan and Afghanistan (Table 1, Fig. 1c–e). In all these patients, the variant was detected by clinical ES and all parents are asymptomatic heterozygotes. Patients 4 and 5 were born to double first-cousin parents from Pakistan. Both are nonambulatory and nonverbal, with dystonic movements and epilepsy appearing within the first few months of life. At age 1.5 years, patient 4’s brain MRI displayed nonspecific T2 signal hyperintensity in the periventricular white matter and globi pallidi, right sided mesial temporal sclerosis, thinned corpus callosum, underopercularization of the Sylvian fissures, small optic nerves, and delayed myelination. At age 2, patient 5’s MRI revealed volume loss in the cortex, cerebellum, and frontal lobe (Fig. 1f). Three other individuals in this family were reported to have had neurological diseases of unknown cause, but additional details were unavailable.

Patient 6 is a girl born to parents from Afghanistan with no reported consanguinity. She has poor weight gain, microcephaly, esotropia, and infantile spasms with onset at age 3 months (Table 1). At age 2, the child can bring her hands to her mouth but cannot reach for or hold objects. She is unable to roll, sit, or stand and has no speech. A brain MRI at age 2 revealed delayed myelination and mild cerebellar volume loss (Fig. 1f).

Patients 7 and 8 are sibling girls in a family from Afghanistan with no reported consanguinity. These two patients had infantile spasms before 4 months, hypotonia, and severe intellectual impairment (Table 1). The older sibling (patient 8, III.7 on the pedigree) can move her arms and legs independently but cannot sit or crawl. The younger (patient 7, III.8) is not able to roll over. They also had an older sister (III.4) who died at age 8 with a similar disorder and was never able to crawl. In patient 7, a brain MRI at age 2 months revealed mild-to-moderate prominence of the cortical cerebrospinal fluid (CSF) space but age-appropriate myelination and no other abnormalities (Fig. 1f). MR spectroscopy in this patient revealed normal lactate but some voxels with low N-acetylaspartate to choline and N-acetylaspartate to creatine ratios.

**Fig. 2:**
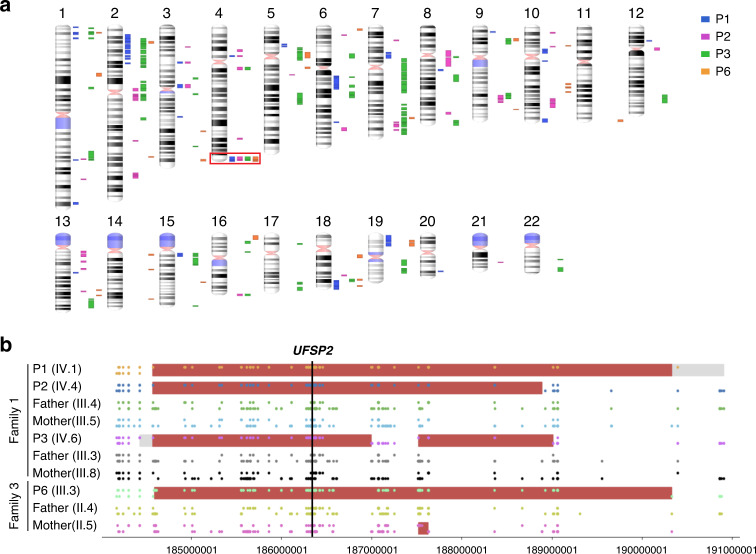
Runs of homozygosity analysis in families 1 and 3. (**a**) Chromosomal distribution of homozygous regions in patients P1, P2, P3, and P6. The displayed regions are larger than 1 Mb and are homozygous in the patients but not the parents. The sole homozygous region shared by all four patients is indicated by the red frame on chromosome 4. (**b**) Schematic of homozygous segments on chromosome 4q in families 1 and 3. For each individual, the top line displays markers with homozygous genotypes and the bottom line displays markers with heterozygous genotypes. The homozygous regions are highlighted in color blocks: red for regions common to more than one individual, gray for regions unique to one individual. The *UFSP2* locus is indicated.

This *UFSP2* variant is rare, although its allele frequency is higher in South Asians (MAF = 0.00089) compared with non-Finnish Europeans (MAF = 0.000035) and other populations in the gnomAD database (v2.1.1). Given that many families in Pakistan and Afghanistan belong to the Pathan ethnic group, homozygosity for this variant in our subjects might suggest shared ancestry and enhanced autosomal homozygosity among the affected families. We performed ROH analysis to evaluate the genome-wide homozygosity in patients and parents of families 1 and 3, where complete ES data were readily available. Comparing homozygous genomic regions identified in each patient (P1, 2, 3, and 6) but not in the parents revealed two adjacent stretches of homozygosity measuring 2.4 and 1.7 Mb on chromosome 4q. The *UFSP2* locus is within the 2.4 Mb region (Fig. 2a,b and Table S1). None of the parents are homozygous at the *UFSP2* locus, although the mother of patient 6 (II.5 in Fig. 1d) does contain a small region of homozygosity within the 1.7 Mb block (Fig. 2b). Clinical ES data were not readily available for families 2 and 4, so we do not know whether the patients in these families share the same haplotype as those in families 1 and 3.

The cosegregation of *UFSP2* p.V115E with the disease in multiple families constitutes strong evidence for its pathogenicity. The probability (*N*) that genotype–phenotype cosegregation occurred by chance is (1/4)^3^ × (3/4)^2^ ≈ 0.009, where the first factor corresponds to the three affected sibpairs in families 1, 2, and 4, and the second factor corresponds to the unaffected siblings in families 1 and 3. This is lower than the recommended criterion of *N* ≤ 1/16 (0.06) for strong evidence of pathogenicity.^20^ Note that in family 1 we took a conservative approach by assuming that the most recent common ancestor of the rs142500730[T] allele was before generation I, and therefore did not include patient 3 in the calculation. With this evidence for pathogenicity, we proceeded to functional analysis of the variant.

### Functional analysis of UFSP2 and UFMylation in fibroblasts from affected patients

**Fig. 3:**
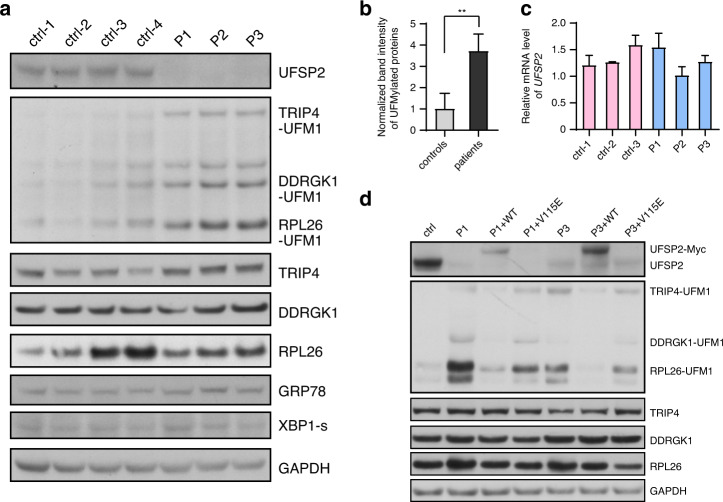
UFSP2 expression and UFMylation marks in patient and control fibroblasts. (**a**) Immunoblot analysis of primary human fibroblasts from four control subjects and patients P1, P2 and P3. (**b**) Quantitative analysis of the immunoblots for UFMylated proteins in Fig. 3a. The intensities for total anti-UFM1 signal were normalized to GAPDH. **p < 0.01. (**c**) Quantitative real-time polymerase chain reaction (RT-PCR) of *UFSP2* messenger RNA (mRNA) in fibroblasts from three control subjects and patients P1, P2, and P3. (**d**) Immunoblot analysis of UFMylated proteins in P1 and P3 fibroblasts ectopically expressing wild-type (WT) or V115E variant of UFSP2.

Skin biopsies were performed in patients 1–3 to establish fibroblast cultures. Immunoblotting of proteins extracted from these cell lines revealed markedly reduced UFSP2 levels relative to cells derived from healthy subjects (Fig. 3a). *UFSP2* messenger RNA (mRNA) levels assessed by quantitative RT-PCR revealed no differences among the cultures, indicating that the effect of the variant was likely not mediated by changes in RNA stability (Fig. 3c). An antibody against UFM1 revealed that the patients’ cells contained enhanced levels of several UFM1-conjugated proteins, including DDRGK1, TRIP4 and RPL26, despite no substantial increases in the total abundance of these proteins (Fig. 3a, b). Ectopic expression of wild-type UFSP2 but not the mutant normalized the levels of UFMylated proteins in patient fibroblasts (Fig. 3d). The ectopically expressed mutant was difficult to detect by immunoblotting, consistent with the V115E variant causing UFSP2 destabilization.

Mammalian UFMylation is carried out by a multiprotein complex predominantly located at the cytosolic side of the ER membrane, and the pathway is involved in vesicular trafficking and ER homeostasis.^21–23^ Disruption of UFMylation pathway induces ER stress and activates the unfolded protein response in mouse hematopoietic stem cells^24,25^ and cardiomyocytes.^26^ However, no activation of ER stress was detected in the three UFSP2-mutant fibroblast lines as assessed by immunoblotting for the ER stress markers GRP78 and spliced XBP1 (Fig. 3a).

### *UFSP2* expression and structural aspects of disease-causing variants

**Fig. 4:**
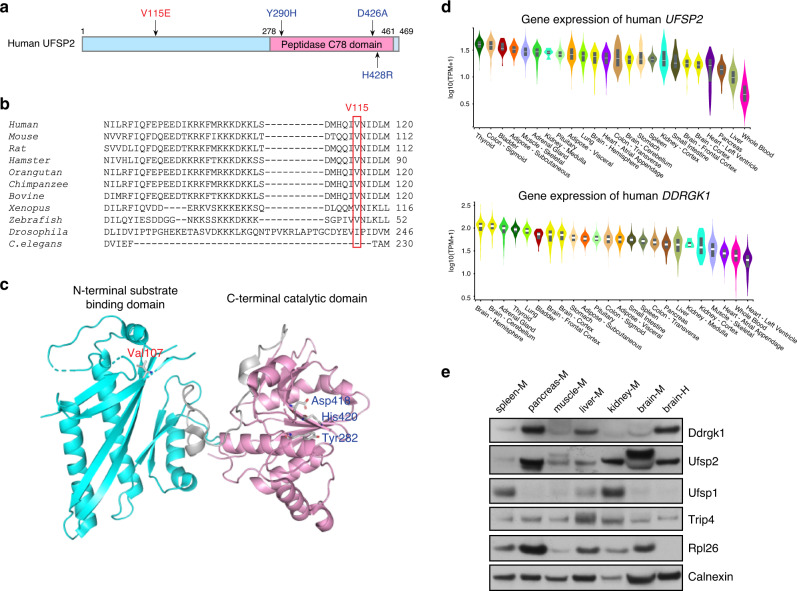
Structural analysis of human UFSP2 variants and expression of UFSP2 and its targets in human and mouse tissues. (**a**) Schematic of human UFSP2 functional domains. The patient-derived variants are indicated, with those causing dominantly inherited disease in blue and the new variant causing recessively inherited disease in red. (**b**) Conservation of the V115 residue (red frame) across multiple species. (**c**) Three-dimensional structure of mouse Ufsp2. The homologous residues mutated in patients are indicated. Val107, Tyr282, Asp418, and His420 correspond to Val115, Tyr290, Asp426, and His428 respectively, in human UFSP2. (**d**) Gene expression of *UFSP2* and *DDRGK1* in 24 human tissues from the Genotype–Tissue Expression (GTEx) database. (**e**) Immunoblots showing expression of Ufsp2 and its targets, Ddrgk1, Trip4, and Rpl26, in mouse (M) tissues and human (H) brain. Calnexin is used as a loading control.

Although disease-causing *UFSP2* variants have been reported in humans, these diseases involve skeletal anomalies rather than neurological dysfunction. Patients with autosomal dominant Beukes hip dysplasia have a Y290H variant in UFSP2.^12^ Spondyloepimetaphyseal dysplasia, Di Rocco type (SEMDDR) occurred in an Italian family with a D426A variant in UFSP2^11^ and a Chinese patient with a H428R variant.^13^ None of the reported patients with these diseases had neurological impairments or seizures. Y290, D426, and H428 are located in UFSP2’s C-terminal C78 peptidase domain required for its catalytic activity (Fig. 4a), whereas V115 is within the N-terminal domain that interacts with DDRGK1, an ER-localized UFMylation target.^9^ V115 is highly conserved across species, including all vertebrates studied (Fig. 4b). Three-dimensional structural analysis of the mouse homolog (Protein Data Bank [PDB]: 3OQC) revealed that the N-terminal region of Ufsp2 consists of mixed α-helices and β-strands.^27^ The homologous Val in mouse Ufsp2 (V107) is located at the β3 strand and in the core of the protein–protein interacting domain (Fig. 4c). Mutating Val to Glu introduces a larger, less hydrophobic and negatively charged residue into the β-sheet structure, a change predicted to impair the local hydrophobic interactions. Human UFSP2 Y290, D426, and H428 are equivalent to mouse Ufsp2 Y282, D418, and H420 (Fig. 4c), which contribute to the catalytic core in the active site of the mouse protein.^27^ Mutating Y282 to His inactivated Ufsp2’s catalytic activity.^27^ These data suggest that variants observed in human skeletal dysplasias impact UFSP2’s catalytic activity, but the V115E variant in patients with neurological disorders may have other effects including reduced protein stability and possibly reduced interaction with UFMylated targets.

To obtain insights into UFSP2’s relevance to neurological disease, we examined the expression of *UFSP2* and several UFMylation targets across mouse and human tissues. According to the Genotype–Tissue Expression (GTEx) database, *UFSP2* and *DDRGK1* mRNAs are expressed in multiple regions of the human brain (Fig. 4d). Immunoblotting confirmed expression of UFSP2 and DDRGK1, as well as the UFMylation target TRIP4, in human brain (Fig. 4e). Ufsp2, Ddrgk1, Trip4, and the UFMylation target Rpl26 were also observed in the mouse brain, although Ddrgk1 was much less abundant in the brain than in mouse pancreas and liver.

## DISCUSSION

Pediatric neurodevelopmental syndromes involving epilepsy are genetically heterogeneous. Although ES increasingly identifies genomic variants in patients with these diseases, establishing the pathogenicity of such variants is challenging. Using guidelines from the American College of Medical Genetics and Genomics and Association for Molecular Pathology, together with the additional information provided by cosegregation analysis,^20,28^ the *UFSP2* c.344T>A (p.V115E) variant qualifies as pathogenic based on the following criteria: (1) cosegregation probability less than 1/16 in an analysis involving multiple families, (2) functional studies supporting a damaging effect on the gene product, (3) extremely low frequency in gnomAD (overall MAF = 0.00013), and (4) detected in *trans* as a recessive allele. Criteria 1 and 2 are considered strong evidence for pathogenicity, while criteria 3 and 4 are considered moderately supportive of pathogenicity; together these data meet the threshold for pathogenicity. The structural and evolutionary analysis of UFSP2, its expression in the relevant tissues, and the lack of other shared, potentially disease-causing alleles among the families in the study, further support the variant’s pathogenicity in the neurologic disease observed in our patients.

Ample evidence from human genetics indicates that UFMylation is important in brain development, as several components of the pathway are mutated in autosomal recessive diseases affecting the central nervous system. Similar to the patients described here, *UFM1* variants cause a disorder of severe intellectual disability, intractable epilepsy, microcephaly, and poor growth (leukodystrophy, hypomyelinating, 14, HLD14, OMIM 617899). This disease has been described in families of Roma and Sudanese descent.^29,30^ *UBA5* variants cause a subtype of early infantile epileptic encephalopathy, with fibroblasts from the affected individuals suggesting dysfunctional E1-like activity in the mutants (EIEE44, OMIM 617132).^31,32^ *UBA5* variants have also been reported in individuals with an autosomal recessive form of spinocerebellar ataxia (SCAR24, OMIM 617133).^33^ Variants in *UFC1*, which encodes the E2 component, cause an autosomal recessive neurodevelopmental disorder with spasticity and poor growth (NEDSG, OMIM 618076).^30,34^

Our study connects *UFSP2* to a phenotype with overlapping features, providing further evidence for the importance of UFMylation in human brain development. We demonstrate that UFSP2 and at least one UFMylated protein, DDRGK1, are expressed in the human brain. Previously reported defects in *UFM1* and in the E1 and E2 components of the pathway were associated with decreased function of the UFMylation cascade,^30–32^ suggesting that the pathology involves an interruption in UFMylation-dependent mechanisms of target protein function. Reduced UFSP2 expression in fibroblasts indicates a loss of function effect for the V115E variant, and we anticipated that these cells would also display reduced UFMylation. The increased levels of UFMylated targets in these cells suggest that the variant more prominently affects de-UFMylation rather than UFMylation. Reconstituting fibroblasts with wild-type UFSP2 reduced UFMylation marks, also indicating defective de-UFMylation in the patients’ cells. We do not know how the variant affects UFMylation in the brain, but we speculate that UFSP1 or other enzymes compensate for pro-UFM1 cleavage in some tissues, thereby allowing UFMylation to occur even in the context of hypomorphic UFSP2 variants. The functions of UFSP1 and UFSP2 are at least partially redundant, as in vitro assays have demonstrated that both UFSP1 and UFSP2 can cleave pro-UFM1 and release UFM1 from UFMylated proteins.^6^ However, while these proteins share sequence similarity in their C-terminal catalytic domains, UFSP2 is more than twice as large. The N-terminal domain that contains V115 and appears to promote associations with UFMylated proteins is unique to UFSP2. This may explain how variants in this region result in excess UFMylation of at least some targets. Our data also suggest that V115 is required for protein stability, perhaps through substrate binding, because homozygosity for V115E results in reduced UFSP2 abundance.

Along these lines, a curious aspect of the *UFSP2* variant described here is that the V115E substitution results in central nervous system dysfunction but no obvious skeletal anomalies, while other *UFSP2* variants result in autosomal dominant skeletal dysplasias but no seizures or defects in intellectual development.^11–13^ These previous reports, along with the observation of *DDRGK1* variants in an autosomal recessive skeletal disorder (spondyloepimetaphyseal dysplasia, Shohat type, OMIM 616177) provide convincing evidence that UFMylation is required in the human skeletal system. These *UFSP2* variants are localized within the peptidase domain, unlike the V115E variant described here. The variant in Beukes hip dysplasia reduces UFSP2’s in vitro catalytic activity, although its effects on the levels of UFMylated proteins in cells is unknown.^12^ Understanding the pathophysiology of these *UFSP2*-related diseases will require a more comprehensive assessment of how each variant alters the UFMylated proteome in relevant tissues, and how these changes impact the function of UFMylated proteins.

## Supplementary information

Supplementary Information

Supplementary tableS1

## Data Availability

The materials and protocols used in this study are available to share upon request. The *UFSP2* variant (c.344T>A; p.V115E) has been deposited in ClinVar with the accession number SCV001338803.
