# Supplementary material for: A pathogenic UFSP2 variant in an autosomal recessive form of pediatric neurodevelopmental anomalies and epilepsy
Source: Genet Med. 2021 Jan 20;23(5):900–8. doi: 10.1038/s41436-020-01071-z (PMC8105169; doi:10.1038/s41436-020-01071-z)
Supplement: Supplementary file 1 — Supplementary Information [file 41436_2020_1071_MOESM1_ESM.docx]

**Supplemental Information**

**Table S1.** **Runs of homozygosity (>1Mb) in patients and parents from families 1 and 3.** Homozygous regions of 1 Mb or greater in length in patients and parents of families 1 and 3. A “0” indicates that the region is not present in the individual, and a “1” indicates that the region is present.
